# Engineering Red Blood Cell Membrane-Coated PLGA Nanoparticles for Kahweol Delivery: Formulation Development and Pharmacokinetic Assessment

**DOI:** 10.3390/molecules31183239

**Published:** 2026-09-14

**Authors:** Okan Ali Aksoy, Yagmur Okcay, Alperen Enes Solmaz, Kübra Kılıç, Berk Alp Göksel, Burcu Eser, Özgür Eşim, İsmail Mert Vural, Ayhan Savaşer, Yalçın Özkan

**Affiliations:** 1 Gulhane Institute of Health Sciences, University of Health Sciences, Ankara 06018, Türkiye; berkalp.goksel@sbu.edu.tr (B.A.G.); burcu.eser@sbu.edu.tr (B.E.); ozgur.esim@sbu.edu.tr (Ö.E.); yalcin.ozkan@sbu.edu.tr (Y.Ö.); 2 Department of Pharmacology, Gulhane Faculty of Pharmacy, University of Health Sciences, Ankara 06018, Türkiye; yagmur.okcay@sbu.edu.tr (Y.O.); ismailmert.vural@sbu.edu.tr (İ.M.V.); 3 Department of Pharmaceutical Technology, Gulhane Faculty of Pharmacy, University of Health Sciences, Ankara 06018, Türkiye; alperensolmaz006@gmail.com (A.E.S.); ayhan.savaser@sbu.edu.tr (A.S.); 4 Department of Pharmaceutical Biotechnology, Gulhane Faculty of Pharmacy, University of Health Sciences, Ankara 06018, Türkiye; kubra.kilic@sbu.edu.tr

**Keywords:** kahweol, pharmacokinetic, PLGA, RBC coating

## Abstract

Kahweol is an active diterpene with anti-inflammatory, antioxidant, and anticancer properties; however, its use may be limited by unfavorable pharmacokinetic characteristics. This study aimed to develop kahweol-loaded poly(lactic-co-glycolic acid) (PLGA) and red blood cell membrane-coated PLGA (RBC-PLGA) nanoparticles and evaluate their in vitro release behavior and in vivo pharmacokinetic profiles. Kahweol-loaded PLGA nanoparticles were prepared using the emulsification-solvent evaporation method and subsequently coated with rabbit erythrocyte membranes, a type of blood cell membrane, to obtain RBC-PLGA nanoparticles. Particle size, zeta potential, morphology, and encapsulation efficiency were characterized. In vitro release studies were performed using the dialysis bag method. Pharmacokinetic profiles of free kahweol, kahweol-loaded PLGA, and kahweol-loaded RBC-PLGA nanoparticles were evaluated in rabbits following intravenous administration (0.5 mg/kg), and plasma kahweol concentrations were analyzed by LC-MS/MS. PLGA and RBC-PLGA nanoparticles showed high encapsulation efficiency (>90%) and sustained biphasic release compared with the rapid burst release of free kahweol. Pharmacokinetic analysis demonstrated that PLGA and RBC-PLGA nanoparticles reduced peak plasma concentrations and prolonged systemic exposure. Among the nanoparticles, RBC-PLGA exhibited the most prolonged pharmacokinetic profile, with delayed time to maximum plasma concentration (T_max_), extended half-life, and increased overall exposure. The nanoparticles improved the pharmacokinetic profile of kahweol by enabling sustained release and prolonged systemic exposure, supporting their potential as delivery platforms for future applications.

## 1. Introduction

**Figure 1:**
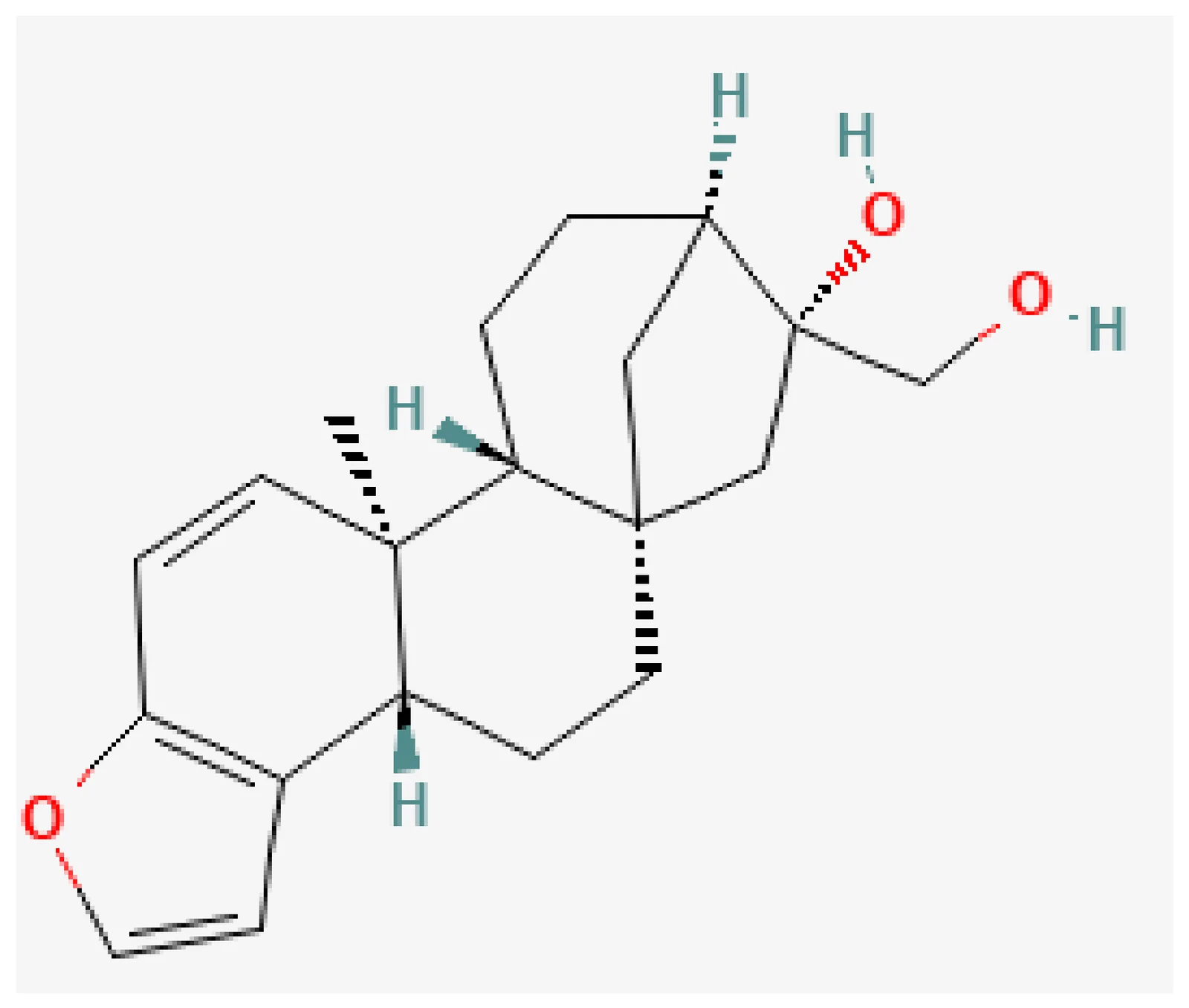
Chemical structure of kahweol (PubChem CID: 114778).

Kahweol (C_20_H_26_O_3_; molecular weight: 314.42 g/mol) is a naturally occurring pentacyclic diterpene alcohol belonging to the ent-kaurane family [1]. The chemical structure of kahweol, one of the major diterpenes found in the lipid fraction of coffee, is shown in Figure 1. In coffee beans, kahweol occurs predominantly in forms esterified with various fatty acids, such as palmitic and linoleic acids, while a smaller proportion is present as the free diterpene. Although kahweol has been reported to occur at particularly high levels in *Coffea arabica* L., its presence has also been demonstrated at lower levels in *Coffea canephora* (Robusta) and various wild Coffea species. Therefore, *Coffea arabica* is considered one of the principal and richest commercial sources of kahweol. In in vivo inflammatory models, kahweol has been shown to reduce inflammatory markers, including cyclooxygenase-2 (COX-2), inducible nitric oxide synthase (iNOS), tumor necrosis factor (TNF-α), and prostaglandin-E2 (PGE2) [2,3,4,5,6,7,8]. Anticancer studies have shown that kahweol can inhibit tumor-cell proliferation and clonogenicity and promote apoptotic signaling [9]. Once ingested, approximately 70% of kahweol is absorbed in the small intestine, subsequently accumulating in the liver and gastrointestinal tract through the enterohepatic cycle. While the body attempts to metabolize this lipophilic compound into water-soluble products via liver pathways like glucuronidation and sulphation to facilitate excretion, research shows that only about 1% or less is detected in urine, suggesting that a major part of absorbed kahweol undergoes more extensive metabolism [10]. Despite its promising biological effects, the therapeutic application of kahweol may be limited by unfavorable pharmaceutical and pharmacokinetic properties. According to the product information provided by Cayman Chemical, kahweol is sparingly soluble in aqueous media, with a reported solubility of approximately 0.02 mg/mL in ethanol: phosphate buffer serum (1:50, pH 7.2), which may adversely affect its formulation, systemic distribution, and bioavailability. In addition, rapid elimination and insufficient circulation time may restrict the maintenance of therapeutically relevant concentrations in vivo. PLGA is one of the most widely used biodegradable and biocompatible polymers in drug delivery research [11]. PLGA-based nanoparticles have been extensively investigated because they can encapsulate hydrophobic compounds, protect them from premature degradation, and provide controlled or sustained drug release. Such systems may also modify the absorption, distribution, and elimination characteristics of loaded agents, thereby improving their overall pharmacokinetic performance [12]. Biomimetic nanoparticles have recently emerged as a promising drug delivery strategy. RBC membrane-coated nanoparticles are attractive because erythrocytes naturally circulate for prolonged periods and possess membrane components that reduce phagocytic clearance [13]. By combining the controlled-release properties of PLGA with the immune-evasive characteristics of RBC membranes, RBC-PLGA nanoparticles may offer additional pharmacokinetic advantages over conventional PLGA formulations. Such a system may be especially valuable for compounds like kahweol, for which prolonged circulation and sustained exposure are desirable. The aims of this study were to develop kahweol-loaded PLGA and RBC-PLGA nanoparticles and to evaluate their encapsulation efficiency, in vitro release behavior, and in vivo pharmacokinetic profiles in rabbits.

## 2. Materials and Methods

### 2.1. Materials

CorbionPLGA-Purasorb^®^ poly(D,L-lactide-co-glycolide) (PDLG) 5010 (50:50) was kindly supplied as a gift sample by Corbion Purac (Gorinchem, The Netherlands). Kahweol (≥95% purity, Item No. 14015) was purchased from Cayman Chemical (Ann Arbor, MI, USA). Polyvinyl alcohol (PVA) (Mowiol 4-88), dialysis bag, dichloromethane, formic acid, acetonitrile and disodium ethylenediaminetetraacetic acid (EDTA) were obtained from Sigma-Aldrich, Darmstadt, Germany. All reagents used in this study were of analytical grade and were employed without further purification.

### 2.2. Preparation of Kahweol-Loaded PLGA Nanoparticles

Kahweol-loaded PLGA nanoparticles were prepared using the emulsification–solvent evaporation method. Briefly, 100 mg of PLGA (PDLG 5010, Gorinchem, The Netherlands) and 3 mg of kahweol were dissolved in 3 mL of dichloromethane. The organic phase was then gradually added to 10 mL of 1% (*w*/*v*) aqueous PVA solution and emulsified. The resulting emulsion was sonicated in an ice bath using a Bandelin Sonopuls probe sonicator (Bandelin electronic GmbH & Co. KG, Berlin, Germany) equipped with a TT19 sonotrode (19 mm diameter) at 50 W for 60 s in continuous mode. Following sonication, dichloromethane was removed by rotary evaporation at 37 °C under a vacuum of 150 mbar for 10 min [14]. The resulting nanoparticle suspension was purified by three successive centrifugation steps at 10,000 rpm for 15 min at 4 °C. The purified nanoparticles were subsequently frozen at −80 °C and lyophilized.

### 2.3. Preparation of RBC Ghosts

RBC ghosts were prepared using a hypotonic lysis and extrusion method as previously described [15]. Briefly, fresh heparinized rabbit whole blood was collected and centrifuged at 700× *g* for 10 min at 4 °C to separate plasma, leukocytes, and platelets. The isolated erythrocytes were washed three times with ice-cold 1× PBS containing 1 mM EDTA·2Na. The purified RBCs were then lysed in 0.2 mM EDTA·2Na solution and centrifuged at 20,000× *g* for 10 min at 4 °C. After removal of the supernatant, the pellet was washed again with 0.2 mM EDTA·2Na solution to further purify the RBC membranes. The resulting pink RBC ghost pellet was collected and stored at −80 °C until further use.

### 2.4. Preparation of RBC-PLGA Nanoparticles

Kahweol-loaded RBC-PLGA nanoparticles were prepared by coating PLGA nanoparticles with RBC membranes. In total, 1 mg of kahweol-loaded PLGA nanoparticles was combined with RBC ghosts isolated from 100 μL of whole blood. The core-to-membrane ratio was selected according to a previously reported protocol [16]. The resulting suspension was then passed ten times through sequential 400- and 200-nm polycarbonate membranes using a mini extruder (Avanti Polar Lipids, Alabaster, Al, USA).

### 2.5. Characterization of Nanoparticles

#### 2.5.1. Particle Size Distribution, Zeta Potential, and Morphology

The average particle size and polydispersity index (PDI) of the nanoparticles were determined by photon correlation spectroscopy (PCS) using a Nicomp Nano Z3000 instrument (PSS, Port Richey, FL, USA). Prior to analysis, samples were diluted with ultrapure water and measured at 25 °C with a scattering angle of 90°. The reported particle size represents the mean of three independent measurements, and the results are expressed as mean ± standard deviation from at least three separate nanoparticle batches [17].

The zeta (ζ) potential of the nanoparticles was also measured using the Nicomp Nano Z3000 (PSS, Port Richey, FL, USA). Diluted nanoparticle suspensions were transferred to the measurement cell, which was positioned to coincide with the previously established stationary layer. Measurements were performed under an applied electric field of 10 V.

The loading efficiency of the prepared PLGA and PLGA-RBC nanoparticles was determined indirectly using the developed LC-MS/MS method. Each nanoparticle formulation was prepared as three independent batches (*n* = 3) using the standardized preparation method under identical experimental conditions. Particle size, size distribution, zeta potential, and loading efficiency were determined for each independently prepared batch, and the results were expressed as mean ± standard deviation (SD).

Nanoparticle morphology was examined by transmission electron microscopy (TEM) using a JEOL 2100F RTEM (Akishima, Tokyo, Japan). A 10 μL aliquot of the nanoparticle suspension was deposited onto Formvar-coated copper grids, allowed to dry, and subsequently imaged by TEM [18].

#### 2.5.2. LC-MS/MS Method Development

LC-ESI-MS/MS analysis was performed in triplicate (n = 3) using an Agilent 6420 LC-MS/MS system (Agilent Technologies, Santa Clara, CA, USA) equipped with an ACE C18 analytical column (50 × 2.1 mm, 5 μm). A 20 μL sample was injected for each analysis, while 2 μL was introduced into the mass spectrometer. Chromatographic separation was achieved using a binary gradient elution at a flow rate of 400 μL/min. The column temperature was maintained at 30 °C, whereas the drying gas temperature was set to 350 °C. Additional mass spectrometric parameters included a drying gas flow of 10 L/min, a nebulizer pressure of 40 psi, and a capillary voltage of 1500 V. Detection was carried out by electrospray ionization in multiple reaction monitoring (MRM) mode. Quantification was based on the integrated peak area of the most abundant product ion, while the remaining product ions served as qualifier ions. The optimized MRM transitions and corresponding mass spectrometric parameters are summarized in Table 1.

The mobile phase consisted of water containing 0.01% formic acid (solvent A) and acetonitrile containing 0.01% formic acid (solvent B). Chromatographic separation began with an isocratic elution at 40% solvent B for 0.5 min, followed by a linear gradient to 98% solvent B over 3 min. This composition was maintained until 3 min, after which the mobile phase was returned to 40% solvent B within 0.5 min. The column was then re-equilibrated under the initial conditions for an additional 6 min before the next injection [19].

### 2.6. In Vitro Release Studies

In vitro release studies were performed using the dialysis bag method. Free kahweol, kahweol-loaded PLGA nanoparticles, and PLGA-RBC nanoparticles were prepared in three independent replicates (n = 3). All groups were prepared to contain an equivalent amount of kahweol, suspended in a final volume of 1 mL, and placed into dialysis membranes (molecular weight cut-off: 12–14 kDa). The amount of kahweol was selected to ensure sink conditions throughout the release study. The dialysis membranes containing the samples were immersed in 20 mL of saline phosphate buffer (pH 7.4), which served as the release medium, and the in vitro release studies were performed. The release studies were conducted at 37 °C with a stirring rate of 400 rpm. At predetermined sampling times, 1 mL of the release medium was withdrawn, filtered, and immediately replaced with an equal volume of fresh PBS to maintain sink conditions [20]. The amount of kahweol removed during each previous sampling was considered when calculating the cumulative amount of kahweol released. The concentration of released kahweol was quantified by LC-MS/MS.

To characterize the in vitro drug release behaviour, the experimental release data were fitted to several mathematical kinetic models, including zero-order, first-order, Higuchi, and Korsmeyer–Peppas models. Kinetic modelling was investigated through the DDSolver program [21]. The zero-order model describes drug release at a constant rate that is independent of the drug concentration remaining in the formulation. In contrast, the first-order model assumes that the release rate is concentration-dependent, with the rate of drug release decreasing as the amount of drug remaining in the system decreases. The Higuchi model describes drug release from insoluble or matrix-based systems as a time-dependent diffusion process, in which the cumulative amount of drug released is proportional to the square root of time, primarily based on Fickian diffusion. The Korsmeyer–Peppas model provides an empirical mathematical relationship for characterizing drug release from polymeric delivery systems and enables the predominant release mechanism to be inferred from the release exponent (n).

#### 2.6.1. Determination of Pharmacokinetic Parameters in Rabbits

Six-month-old female New Zealand rabbits were obtained from the Gülhane Animal Center of the University of Health Sciences. The animals were housed under controlled environmental conditions (22 ± 1 °C, 60 ± 10% relative humidity, and a 12 h light/dark cycle) with unrestricted access to standard laboratory chow and water. All experimental procedures complied with the Guidelines for Animal Experimentation of the University of Health Sciences and were approved by the Gülhane Local Ethics Committee (Approval No. 2025/18). Rabbits were administered free kahweol or kahweol-loaded nanoparticle formulations intravenously via the right marginal ear vein at a dose of 0.5 mg/kg. All formulations were prepared at a concentration of 1 mg/mL. Blood samples (0.25 mL each) were collected into heparinized glass capillary tubes from the ear vein at 5, 10, 20, 30, and 45 min, and at 1, 1.5, 2, 3, 4, 6, 8 and 24 h after administration. Plasma was separated by centrifugation at 10,000 rpm for 5 min, transferred to polyethylene tubes, and stored at −40 °C until LC-MS/MS analysis [22].

#### 2.6.2. Statistical Analysis

Pharmacokinetic parameters were calculated using PKSolver 2.0 (China Pharmaceutical University, Nanjing, China). Statistical analyses were performed using GraphPad Prism package program version 10.0.1. Differences among groups were evaluated by one-way analysis of variance (ANOVA) followed by Dunnett’s T3 post hoc test. Data are presented as mean ± SD, and statistical significance was defined as *p* < 0.05.

## 3. Results

**Figure 2:**
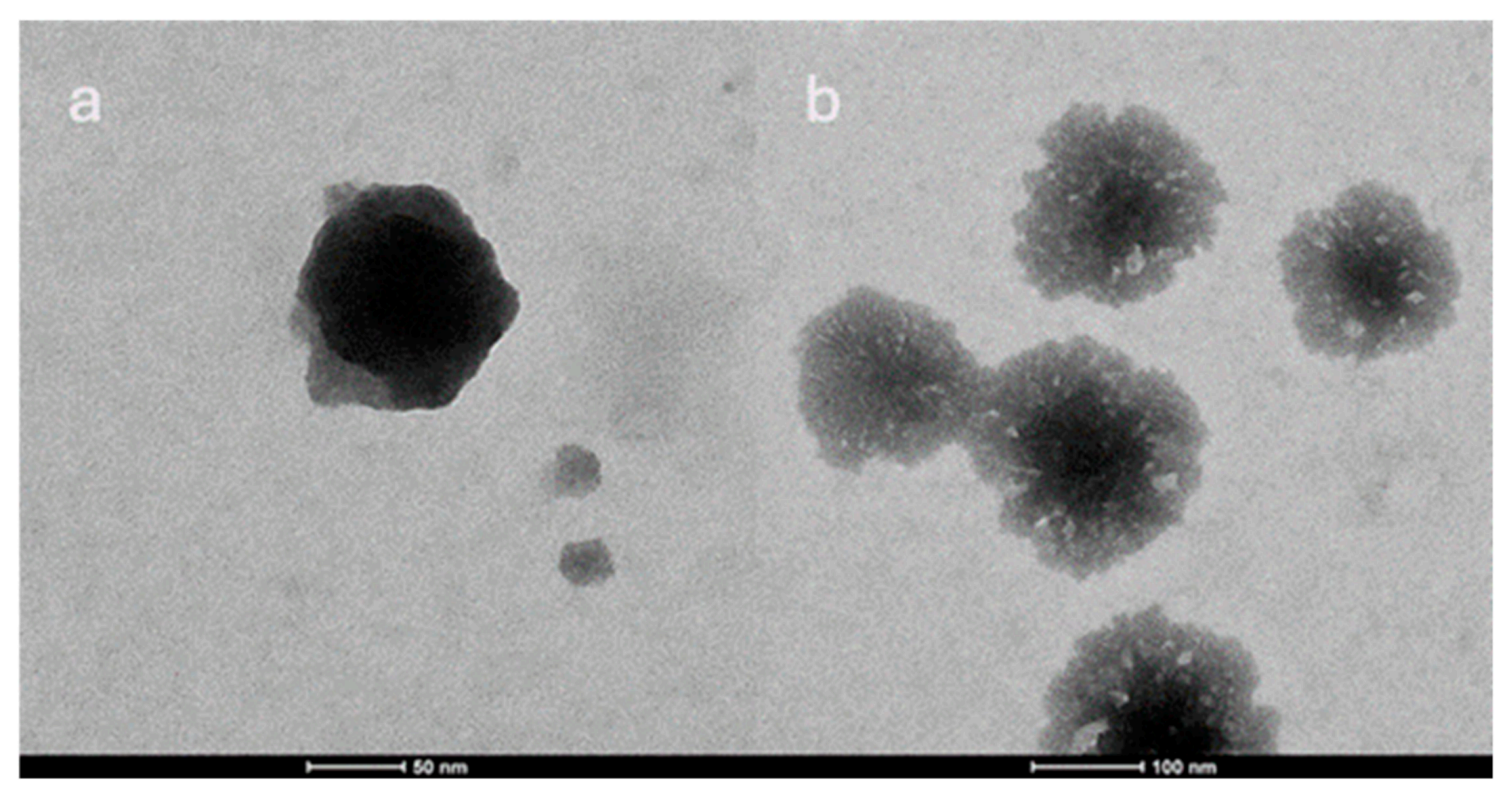
TEM image of PLGA nanoparticles (**a**) and RBC-PLGA nanoparticles (**b**).

The PLGA nanoparticles were prepared by the emulsification-solvent evaporation method, and PLGA nanoparticles were coated with RBCs. The properties of both nanoparticles are shown in Table 2. As shown in Table 2, the size of nanoparticles increased from 297.93 ± 1.52 nm to 315.23 ± 0.09 nm after RBC coating. Moreover, entrapment efficiency decreased from 94.17% to 90.92%, which shows that both nanoparticles showed high encapsulation efficiency, indicating successful incorporation of kahweol into the nanoparticulate systems. Uncoated PLGA nanoparticles had a negative zeta potential due to the presence of uncapped end carboxyl groups of polymers on the particle surface. As shown in Table 2, the zeta potential increased from −23.10 to −16.62 mV in the presence of the adsorbed coating layers of erythrocyte ghosts on the PLGA nanoparticle surface. The morphology of coated RBC-PLGA was observed by TEM in Figure 2. The obtained nanoparticles appeared to be fine, spherical particles with a smooth surface and without any aggregation or adhesion. The PDI shown in Table 2 was low for both nanoparticle systems (0.029 and 0.129 for 297.93 nm and 315.23 nm, respectively) due to a well-proportioned size distribution.

### 3.1. In Vitro Drug Release

**Figure 3:**
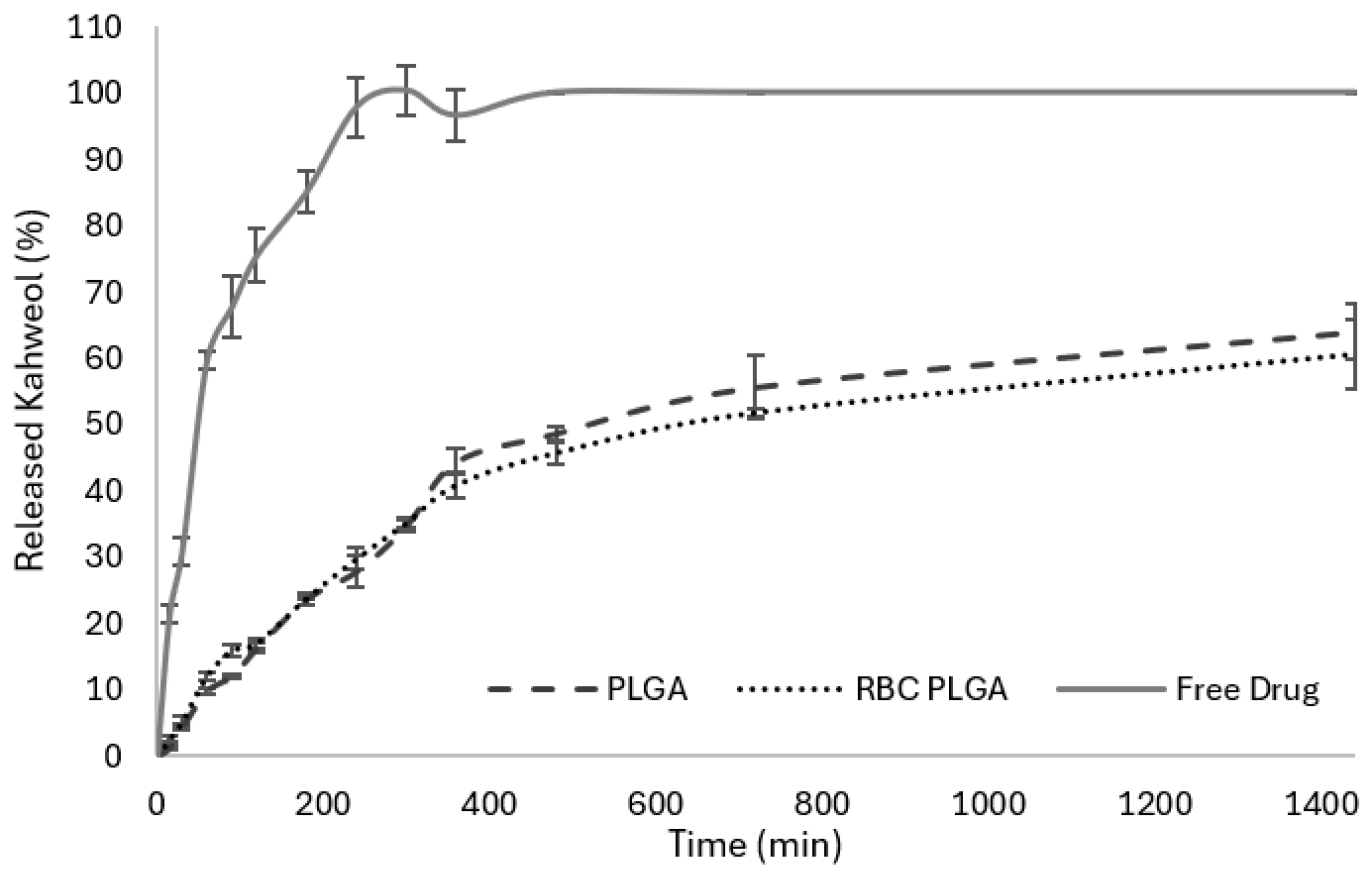
In vitro release profile of kahweol from free drug, PLGA, and RBC-PLGA formulations.

Figure 3 shows that the release profiles of kahweol from PLGA and RBC-PLGA nanoparticles were similar biphasic releases. In the initial burst release phase, 27.91% (PLGA) and 27.81% (RBC-PLGA) of kahweol were released within 4.0 h. The following release was a constant, slower process, and 64.01% (PLGA) and 60.55% (RBC-PLGA) of the cumulative amount of kahweol was released within 24 h. However, the in vitro release study demonstrated clear differences between free kahweol and the formulated nanoparticles (Figure 3). Free kahweol exhibited a rapid release pattern, with approximately 60% released within 60 min and nearly complete release reached by around 240–300 min. In contrast, both PLGA and RBC-PLGA formulations showed a markedly slower and more sustained release profile throughout the experimental period.

Analysis of the in vitro release profiles indicated that the Higuchi model provided a good fit to the release data, suggesting that diffusion plays an important role in the release of the drug from the formulations (Table 3). However, both nanoparticle formulations were also adequately described by the Korsmeyer–Peppas model, although the calculated release exponent (n) differed between the formulations. For the PLGA nanoparticles, the release exponent was n = 0.806, indicating anomalous (non-Fickian) transport. This finding suggests that drug release is governed by a combination of diffusion and polymer relaxation/erosion processes rather than by Fickian diffusion alone. In contrast, the RBC-coated PLGA nanoparticles exhibited a release exponent of n = 1.002. A value of n close to 1 is consistent with Case II transport and approaches zero-order release behavior, indicating a relatively constant release rate over time [23]. These findings suggest that RBC coating may alter the drug-release mechanism and contribute to a more controlled and sustained release profile compared with uncoated PLGA nanoparticles.

### 3.2. Pharmacokinetic Profile of Kahweol and Its Formulations

The plasma concentration–time curves showed marked differences between free kahweol and both nanoparticles. Free kahweol exhibited a sharp early peak, reaching very high plasma concentrations immediately after administration, followed by a rapid decline during the early time points. In contrast, both PLGA and RBC-PLGA nanoparticles showed substantially lower initial plasma concentrations and a more gradual concentration–time profile. Compared with free kahweol, the nanoparticles appeared to reduce the initial burst exposure and provide a more sustained systemic presence over time. Among the tested groups, free kahweol produced the highest early plasma concentrations, whereas PLGA and RBC-PLGA maintained relatively stable concentrations at later time points. The RBC-PLGA nanoparticles showed the most prolonged profile, consistent with delayed release and extended circulation.

The differences observed between the particle sizes determined by DLS and TEM may be attributed to the distinct measurement principles and sample preparation procedures of these techniques. DLS determines the hydrodynamic diameter of nanoparticles dispersed in a liquid medium, whereas conventional TEM provides direct visualization of particles after deposition and drying on a grid. Therefore, solvent removal and possible structural changes during TEM sample preparation may influence the apparent particle size. In addition, DLS provides an intensity-weighted measurement and is particularly sensitive to the presence of larger particles or aggregates, whereas TEM provides particle-by-particle information from a limited number of particles within selected microscopic fields. Thus, differences between the particle sizes obtained by DLS and TEM should be interpreted considering both the different physical states of the samples during analysis and the inherent limitations of the two techniques [24].

Table 4 shows the pharmacokinetic parameters of free kahweol, kahweol-loaded PLGA and kahweol-loaded RBC-PLGA nanoparticles in rabbit plasma. Free kahweol showed a pharmacokinetic profile marked by rapid systemic appearance and high initial plasma exposure, followed by a relatively limited duration in circulation. The very short T_max_ (0.083 h) and high maximum plasma concentration (C_max_) (1272.202 ± 40.831 ng/mL) indicate that kahweol reached peak plasma levels within minutes after administration, consistent with a pronounced burst exposure. However, this early peak was not sustained, as reflected by the relatively modest area under the plasma concentration–time curve from time zero to infinity (AUC_0–inf_) (2312.203 ± 395.337 ng/mL*h) and shorter elimination half-life (t_1/2_) (11.608 ± 3.283 h) and mean residence time from time zero to infinity (MRT_0–inf_) (15.778 ± 1.706 h) compared with the formulated groups. The PLGA nanoparticles showed a pharmacokinetic profile characterized by a delayed peak concentration and prolonged systemic retention. The observed T_max_ was 2 h, while the C_max_ (67.930 ± 3.341 ng/mL) and initial plasma concentration (C_0_) (42.401 ± 4.075 ng/mL) values were markedly lower than those of free kahweol. The t_1/2_ (78.735 ± 22.250 h) and observed mean residence time from time zero to infinity (MRT_0–inf_obs_) (116.611 ± 32.150 h) were substantially prolonged, showing that PLGA encapsulation markedly extended the residence time of kahweol in the body. Although the area under the plasma concentration–time curve from time zero to the last measurable concentration (AUC_0–t_) (982.899 ± 35.267 ng/mL*h) was lower than that of free kahweol, the observed area under the plasma concentration–time curve from time zero to infinity (AUC_0–inf_obs_) (5914.464 ± 1722.208 ng/mL*h) was markedly higher, indicating greater total systemic exposure over time. The observed T_max_ of 3 h with the relatively low C_max_ value of 58.652 ± 0.001 ng/mL of RBC-PLGA nanoparticles indicates that these nanoparticles produced a slow-rising and blunted peak plasma profile rather than a sharp early burst. The prolonged t_1/2_ of 99.097 ± 4.306 h and MRT_0–inf_obs_ of 142.920 ± 6.417 h show the prolonged persistence of kahweol in the systemic circulation, supporting sustained systemic retention. Likewise, the high AUC_0–inf_obs_ (6820.672 ± 236.395 ng/mL*h) indicates substantial overall exposure over time despite the moderate peak concentration, suggesting that drug release and disposition were prolonged rather than concentrated in the early phase. The low observed clearance (Cl_obs_) is also consistent with slow elimination of kahweol from the circulation. Based on the statistical comparisons, both the kahweol-loaded PLGA and kahweol-loaded RBC-PLGA nanoparticles showed significant differences (* *p* < 0.05) compared with free kahweol for nearly all evaluated pharmacokinetic parameters, as indicated in the table. Comparison of pharmacokinetic parameters revealed statistically significant differences (^&^ *p* < 0.05) between the PLGA and RBC-PLGA groups in terms of AUC_0–t_, C_max_, and observed clearance.

## 4. Discussion

The RBC coating was a promising method for enhancing the circulation times of nanoparticles [25]. In this study, after coating of PLGA nanoparticles with RBC, an increased particle size was observed, indicating the formation of a coating layer on the particle surface. Erythrocyte ghosts could effectively adsorb to the particles surface due to the polarity of PLGA particles [26]. However, the entrapment efficiency of the RBC-coated particles was decreased compared with that of the uncoated particles. This might be explained by the increase in the steps in the fabrication procedure [25]. Uncoated PLGA nanoparticles had a negative zeta potential due to the presence of uncapped end carboxyl groups of polymers on the particle surface. Moreover, the zeta potential increased after the nanoparticles were coated with RBC. This might be due to the fact that the coating layers of erythrocyte ghosts, as a nonionic shield, create a surface negative charge and move the plane of shear outwards from the particle surface [26].

**Figure 4:**
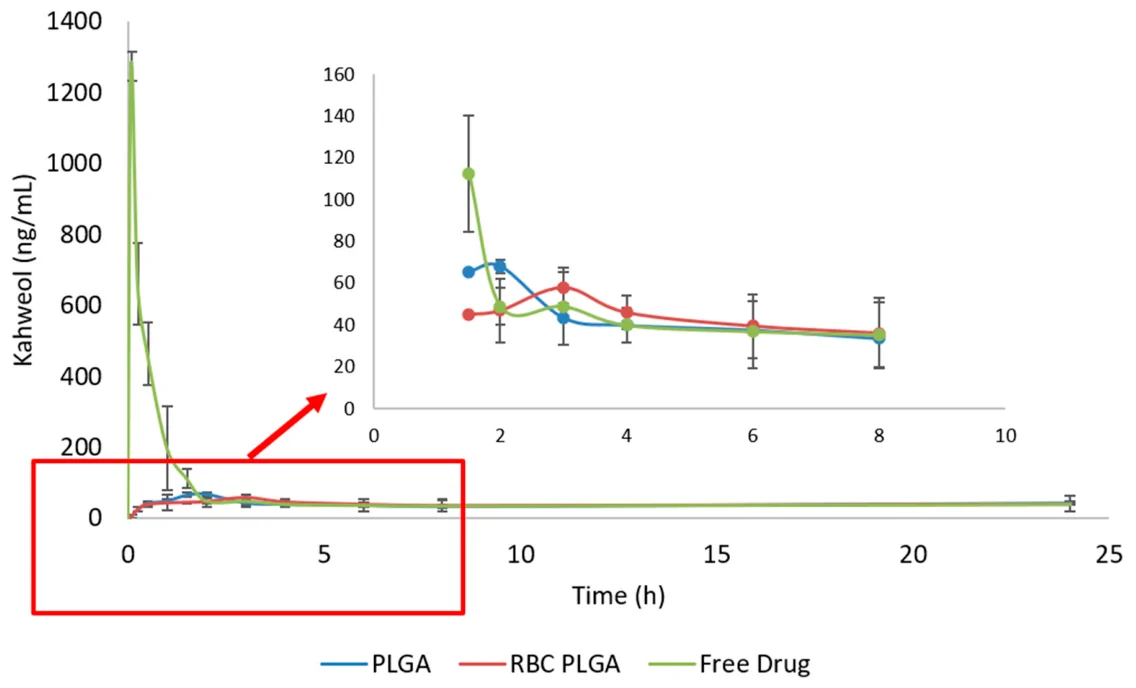
Pharmacokinetic profile of kahweol and its nanoparticles.

As shown in Figure 4, the release profiles of kahweol from PLGA and RBC-PLGA nanoparticles appeared similar biphasic release. The burst release was probably due to a drug that was adsorbed or close to the surface of nanoparticles [27]. A phase of slower release would mainly depend on drug diffusion to the surface of nanoparticles and the PLGA matrix erosion, which was a slower process [28]. The present study demonstrates that formulation markedly influenced both the in vitro release and in vivo pharmacokinetic behavior of kahweol. The in vitro release data showed that free kahweol exhibited a pronounced burst-like release pattern, whereas both PLGA and RBC-PLGA nanoparticles released the compound more gradually over time. Although in vitro release does not directly predict in vivo behavior, this overall directional consistency suggests that encapsulation meaningfully modified kahweol release kinetics rather than simply entrapping the compound.

It is well known that polymer nanoparticles can prolong the drug half-life in the bloodstream, but the time is still short for clinical application due to the rapid uptake of nanoparticles by the Reticuloendothelial System (RES) [28]. Therefore, we have directly investigated the effect of free kahweol and both PLGA and RBC-PLGA nanoparticles on the pharmacokinetics in rabbits after i.v. administration. The pharmacokinetic profile of free kahweol in the present study was marked by rapid entry into the systemic circulation but relatively limited persistence thereafter. The very early attainment of peak plasma levels, together with the high initial concentration observed, points to a pronounced burst-type exposure shortly after administration. Despite this sharp early rise, overall systemic exposure remained comparatively modest, while both persistence in the body and mean residence time were shorter than those observed with the formulated preparations. The results show that free kahweol becomes readily available in the circulation but is cleared or redistributed relatively quickly, thereby limiting sustained exposure over time. This is in line with an in vivo study showing that the effect of free kahweol started in about 5 min after administration and gradually decreased over time [29]. This behavior may represent an important barrier to the practical development of kahweol, as many lipophilic natural compounds, although pharmacologically promising, show restricted systemic persistence and suboptimal disposition. Encapsulation in PLGA altered this disposition profile. Rather than producing the sharp and immediate plasma peak observed with free kahweol, the PLGA nanoparticles generated a slower rise in circulating levels, a markedly attenuated initial peak, and a much more prolonged persistence in the body. Total exposure was also increased, indicating that PLGA not only moderated the early burst phase but also sustained kahweol availability over a considerably extended time window. These findings are consistent with the well-established characteristics of PLGA-based delivery systems. As one of the most widely used biodegradable polymers in controlled drug delivery, PLGA is particularly well suited for hydrophobic compounds, as it can facilitate their incorporation, protect them from premature degradation, and provide sustained release through diffusion- and polymer degradation–mediated processes. Previous studies have shown that PLGA nanoparticles are especially advantageous for poorly water-soluble molecules, improving formulation stability and pharmacokinetic performance by reducing excessive early exposure while maintaining drug availability over a prolonged period [11,30].

Among the tested nanoparticles, the RBC-PLGA system exhibited the most prolonged pharmacokinetic profile. Compared with the PLGA group, the RBC-coated nanoparticle showed significantly lower peak plasma concentrations together with greater overall exposure, indicating that membrane coating further attenuated the early rise in circulating kahweol while enhancing sustained systemic availability. It also displayed longer persistence and residence in the body, consistent with a more durable disposition profile. It has been demonstrated that erythrocyte membrane-camouflaged polymeric nanoparticles can function as long-circulating biomimetic carriers, and subsequent reviews have likewise emphasized their capacity to reduce macrophage uptake and systemic clearance [31,32]. These findings suggest that RBC coating did more than preserve the controlled-release characteristics of PLGA; it likely also altered the in vivo behavior of nanoparticles after administration. The encapsulation findings also support the suitability of these carriers for kahweol delivery. Both nanoparticles exhibited high encapsulation efficiency, indicating effective incorporation of this lipophilic compound into the PLGA-based nanoparticle. This is relevant from a translational perspective, since poor drug loading is a frequent limitation of nanoparticle formulations. Although encapsulation efficiency was slightly reduced in the RBC-coated nanoparticle, it remained markedly high, indicating that the membrane-coating process did not adversely affect kahweol loading.

## 5. Conclusions

The present study shows that nanocarrier-based delivery improved the pharmacokinetic behavior of kahweol. Free kahweol displayed a rapid burst-type profile with very early peak exposure and relatively limited persistence, whereas PLGA encapsulation produced a more sustained-release pattern with prolonged systemic exposure. RBC membrane coating extended this effect further numerically, yielding the longest residence time and highest total exposure among the tested systems. These findings support PLGA and especially RBC-PLGA as promising delivery platforms for kahweol and provide a pharmacokinetic basis for future studies investigating biodistribution, efficacy, and safety in relevant disease models.

## Figures and Tables

**Table 1:** The ion transitions and the settings of the mass analyzer.

Cpd Name	Prec Ion MS1	Prod Ion MS2	Frag (V)	CE (V)	Cell Acc (V)	Polarity
Kahweol	314.9	133	110	26	7	Positive
	314.9	105.1	110	46	7	Positive

**Table 2:** Encapsulation efficiency of the kahweol-loaded PLGA and RBC-PLGA formulations (n = 3).

	Particle Size (±SD) (nm)	PDI (±SD)	Zeta Potential (±SD) (mV)	EE (±SD) (%)	Drug Loading (±SD) (%)
RBC-PLGA	315.23 ± 0.09 *	0.129 ± 0.037	−16.52 ± 2.44	90.92 ± 1.47	4.72 ± 0.38
PLGA	297.93 ± 1.52	0.029 ± 0.012	−23.10 ± 1.41 *	94.17 ± 0.10	4.91 ± 0.12

* *p* < 0.05: significant difference between PLGA and RBC-PLGA groups (n = 3).

**Table 3:** Release kinetics of kahweol-loaded PLGA and kahweol-loaded RBC-PLGA nanoparticles.

	Zero Order		First Order		Higuchi		Korsmeyer-Peppas		
	k	R^2^	K	R^2^	k	R^2^	k	n	R^2^
PLGA	0.112	0.971	1.937	0.890	0.001	0.995	0.316	0.806	0.968
RBC-PLGA	0.107	0.919	1.873	0.934	0.001	0.968	0.113	1.002	0.969

**Table 4:** Pharmacokinetic parameters of free kahweol, kahweol-loaded PLGA and kahweol-loaded RBC-PLGA nanoparticles.

Parameter	Unit	Free Kahweol	Kahweol-Loaded PLGA	Kahweol-Loaded RBC-PLGA
λz	1/h	0.062 ± 0.018	0.009 ± 0.003 *	0.007 ± 0.001 *
t_1/2_	h	11.608 ± 3.283	78.735 ± 22.250 *	99.097 ± 4.306 *
T_max(observed)_	h	0.083	2.000	3.000
C_max_	ng/mL	1272.202 ± 40.831	67.930 ± 3.341 *	58.652 ± 1.491 *^&^
C_0_	ng/mL	1776.089 ± 237.654	42.401 ± 4.075 *	42.978 ± 1.297 *
C_last_obs_/C_max_		0.031 ± 0.017	0.635 ± 0.071 *	0.688 ± 0.014 *
AUC_0–t_	ng/mL*h	1693.990 ± 208.555	982.899 ± 35.267 *	1055.291 ± 14.097 *^&^
AUC_0–inf_obs_	ng/mL*h	2312.203 ± 395.337	5914.464 ± 1722.208	6820.672 ± 236.395 *
AUC_0–t/0–inf_obs_		0.736 ± 0.036	0.173 ± 0.044 *	0.155 ± 0.007 *
AUMC_0–inf_obs_	ng/mL*h^2^	36,818.145 ± 10,182.567	717,378.171 ± 390,981.861	975,565.814 ± 77,550.836 *
MRT_0–inf_obs_	h	15.778 ± 1.706	116.611 ± 32.150 *	142.920 ± 6.417 *
V_z_obs_	(mg)/(ng/mL)	0.008 ± 0.003	0.019 ± 0.001 *	0.021 ± 0.001 *
Cl__obs_	(mg)/(ng/mL)/h	0.0004389 ± 0.000075	0.000085 ± 0.000005 *	0.000146 ± 0.000004 *^&^
V_ss_obs_	(mg)/(ng/mL)	0.007 ± 0.001	0.020 ± 0.001 *	0.021 ± 0.001 *

λz, terminal elimination rate constant; t_1/2_, elimination half-life; T_max_, time to reach maximum plasma concentration; C_max_, maximum observed plasma concentration; C_0_, initial plasma concentration; C_last_obs_/C_max_, ratio of the last observed concentration to the maximum concentration; AUC_0–t_, area under the plasma concentration–time curve from time zero to the last measurable time point; AUC_0–inf_obs_, area under the plasma concentration–time curve from time zero extrapolated to infinity; AUC_0–t_/AUC_0–inf_obs_, fraction of total exposure captured within the observed sampling period; AUMC_0–inf_obs_, area under the first moment curve from time zero to infinity; MRT_0–inf_obs,_ mean residence time from time zero to infinity; V_z_obs_, apparent volume of distribution during the terminal phase; Cl__obs_, observed clearance; and V_ss_obs_, apparent volume of distribution at steady state. * *p* < 0.05: significant difference compared with the free kahweol group, ^&^ *p* < 0.05: significant difference between PLGA and RBC-PLGA groups (n = 4).

## Data Availability

Data will be made available upon reasonable request.
